# Hydroxysafflor Yellow A Attenuates Hydrogen Peroxide-Induced Oxidative Damage on Human Umbilical Vein Endothelial Cells

**DOI:** 10.1155/2020/8214128

**Published:** 2020-11-04

**Authors:** Yuefeng Xie, Yan Guo, ShiDong Cao, Miaomiao Xue, ZhaoYue Fan, ChengXian Gao, Bo Jin

**Affiliations:** ^1^College of Life Science, Zhejiang Chinese Medical University, Hangzhou, Zhejiang 310053, China; ^2^College of Basic Medicine & Public Health, Zhejiang Chinese Medical University, Hangzhou, Zhejiang 310053, China

## Abstract

Oxidative stress of endothelial cells is thought to be a principal cause that induces many cardiovascular diseases. Hydroxysafflor yellow A (HSYA) is a major active component in traditional Chinese medicine safflower and has been used to cure ischemic cardiovascular diseases in China for many years. This study aims to investigate whether HSYA has a repairing effect on oxidative damage of human umbilical vein endothelial cells (HUVECs) induced by H_2_O_2_ and to provide a theoretical basis for the clinical treatment of cardiovascular diseases related to traditional Chinese medicine. Based on the establishment of an H_2_O_2_-induced HUVEC oxidative injury model, the cell viability and proliferation rate were measured by the MTT assay and EdU staining. The intracellular GSH/GSSG ratio and SOD activity were determined by kits. The ROS level was detected by flow cytometry. And the BAX, Bcl-2, PTEN, and AKT expressions were evaluated with western blotting methods. The results showed that HSYA treatment significantly attenuated the H_2_O_2_-induced HUVEC cell damage, increased the intracellular GSH/GSSG ratio and unit SOD activity also, and decreased the intracellular ROS levels. Furthermore, HSYA increased the expressions of AKT and Bcl-2 proteins and inhibited the expressions of BAX and PTEN proteins. These suggest that HSYA exerts repair effects on H_2_O_2_-induced oxidative damage in HUVECs, and the mechanisms may be related to the influence of BAX/Bcl-2 expression and AKT/PTEN signal pathway expression.

## 1. Introduction

Vascular endothelial cells are monolayer of cells located between plasma and vascular tissue, which has emerged as the key regulator of vascular homeostasis [1]. It is well established that endothelial dysfunction is a major determinant of the occurrence and advancement of cardiovascular diseases (CVDs), including atherosclerosis, hypertension, and thrombosis [2–4]. Thus, restoring impaired vascular homeostasis by improving endothelial function can be a promising therapeutic target in the treatment of various CVDs [5]. Several studies have shown that oxidative stress is a vital cause of endothelial dysfunction [6]. Oxidative stress is caused by an imbalance between oxidative and antioxidant mechanisms in the body [7], and excessive accumulation of intracellular redox products has various toxic effects on cells, leading to the occurrence of related diseases [8]. Therefore, endothelial dysfunction caused by oxidative stress is an important factor, leading to vascular damage in metabolism and cardiovascular diseases, and suppression of oxidative stress is considered as a potential strategy for CVD [9, 10].

**Figure 1:**
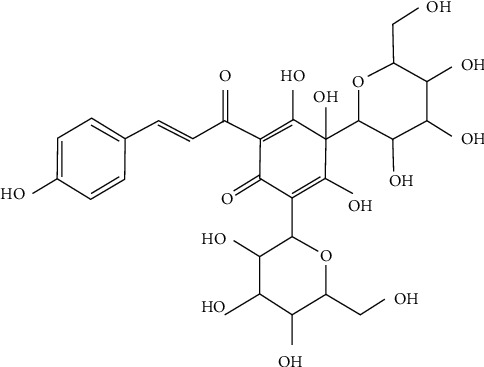
The structure of hydroxysafflor yellow A (HSYA).

In recent years, more and more Chinese medicines and their derivatives were gradually applied to clinical practice with the continuous development of traditional Chinese medicine, and it showed great research value and potential in the treatment of CVD. Hydroxysafflor yellow A (HSYA) is the main active ingredient of *Carthamus tinctorius L.*, which is one kind of the safflower plant, and it is a compound with a single chalcone glycoside structure (Figure 1) [11]. Studies have shown that HSYA (C_27_H_32_O_16_, MW: 612) has anti-inflammatory, antioxidative, and antiangiogenic functions both in vivo and in vitro [12]. However, the molecular mechanisms of HSYA protecting ECs remain unknown. Therefore, in this study, we explored the effect of HSYA on oxidative stress injury in human umbilical vein endothelial cells (HUVECs) and the underlying mechanism.

## 2. Materials and Methods

### 2.1. Materials

Human umbilical vein endothelial cells (HUVECs) cell line was purchased from Cell Bank of Type Culture Academy of Sciences (Shanghai, China). HSYA was purchased from Nanchang Beta Biotechnology Co., Ltd (Jiangxi, China, purity >98%).

### 2.2. Cell Culture

HUVECs were maintained in RPMI 1640 Medium supplemented with 10% fetal bovine serum. Cells were incubated in an incubator with 5% CO_2_ at 37°C with the media and were passage at 80% confluence. The cells were assigned into the following four groups: A, untreated control group; B, H_2_O_2_ (200 *μ*M) model group; C, H_2_O_2_ (200 *μ*M) + HSYA (8 *μ*g/ml) group; D, H_2_O_2_ (200 *μ*M) + HSYA (4 *μ*g/ml) group.

### 2.3. MTT Cell Viability Assay

The MTT assay (Beyotime Institute of Biotechnology, China) was used to evaluate cell viability. In brief, HUVECs were cultured in a 96-well plate (10000 cells/well). After a total of 24 h following plating, cells were pretreated with H_2_O_2_ (200 *μ*M) for 2 h, following which HYSA with different concentrations (8 *μ*g/ml and 4 *μ*g/ml) was added into the plate and cultured at 37°C for a further 24 h. 10 *μ*l MTT was added to each well and incubated at 37°C for 4 h. Subsequently, the culture medium was replaced with 150 *μ*l DMSO. The plate was incubated for 10 min and measured at a wavelength of 490 nm on a microplate reader (Bio Tek, USA). A single group was set up with 6 compound wells, and the results were analyzed statistically.

### 2.4. EdU Cell Proliferation Assay

Following EdU kit (Beyotime Institute of Biotechnology, China) instructions, each treated HUVECs were cultured in a 12-well plate, and 1 ml of EdU medium was added and incubated at 37°C for 2 h; 200 *μ*l of the click staining reaction solution was added and incubated for 30 minutes in the dark, and 200 *μ*l of 1 × Hoechst 33342 reaction solution was incubated for 10 minutes. After washing 3 times with PBS, 5 visual fields were randomly selected under the fluorescence microscope (Nikon, Japan) for observation. Green fluorescent cells are newly proliferated cells, blue fluorescent cells are all cells, and the ratio of the number of green fluorescent cells to blue fluorescent cells represents the cell proliferation rates.

### 2.5. GSH/GSSG Determination

Following GSH/GSSG kit (Beyotime Institute of Biotechnology, China) instructions, collect the cells and add protein to remove the M solution for lysis. Centrifuge at 10,000*g* for 10 min. Take the supernatant. Add the working solution and measure its absorbance at 412 nm with a microplate reader (Bio Tek, USA) to obtain the total glutathione content. Take the above sample. The GSH was cleared, and the measurement wavelength was at 412 nm. The content of the oxidized GSSG was obtained, and the GSH/GSSG ratio was calculated.

### 2.6. Detection of Superoxide Dismutase (SOD)

We used total superoxide dismutase assay kit with WST-8 (Beyotime Institute of Biotechnology, China) to detect the SOD activity in cell lysates. After treatment, cell lysates were prepared, and the protein concentration was measured using the BCA assay (Beyotime Institute of Biotechnology, China). The SOD levels were quantified according to the manufacturer's instructions.

### 2.7. Intracellular ROS Detection

Following the manufacturer's instructions, measure ROS levels in cells using a reactive oxygen analysis kit (Beyotime Institute of Biotechnology, China). In brief, the HUVECs were collected and washed three times with cold PBS. Then, the cells were incubated with the ROS detection work solutions (10 *μ*M) at 37°C for 20 min in the dark and mix up and down every 5 minutes. Use flow cytometry (BD Biosciences, USA) to detect the ROS level of each group.

### 2.8. Western Blotting

After treatments, cells were harvested and washed with cold phosphate-buffered saline (PBS). Cells were lysed with RIPA buffer containing protease and phosphatase inhibitor cocktails and centrifuged. The supernatants were collected and quantified for protein concentration with bicinchoninic acid (BCA) kit (Beyotime Institute of Biotechnology, China) according to the manufacturer's instructions, separated by 10% SDS-PAGE, and transferred to polyvinylidene difluoride membranes (PVDF). The membranes were blocked with 5% BSA in TBS containing 0.1% Tween-20 (TBST) for 2 h at room temperature and then incubated sequentially with primary antibodies at 4°C overnight. A variety of primary antibodies were used during these experiments: BAX (ImmunoWay, YT0459, 1 : 1000), Bcl-2 (ImmunoWay, YM3041, 1 : 1000), AKT (ImmunoWay, YT0185, 1 : 2000), PTEN (Cell Signaling Technology, 9188S, 1 : 1000), and GAPDH (1 : 2000). After primary antibody incubation, the membranes were washed with TBST three times and incubated with either goat anti-mouse or goat anti-rabbit horseradish peroxidase-conjugated secondary antibodies for 2 h. After being washed with TBST three times, the membranes were developed with electrochemiluminescence (ECL) reagent (Biosharp company, BL520A). The density of immunoblotting bands was quantified using Analyzer software.

### 2.9. Statistical Analysis

All assays were independently done three times. All data were presented as the mean±standard error of the mean (SEM). Quantitative data were analyzed by one-way analysis of variance (ANOVA). The Student–Newman–Keuls test was used for post hoc analysis to identify significant differences between groups. Statistical significance was set at *p* < 0.05.

## 3. Results

### 3.1. Effects of H_2_O_2_ and HSYA on the Viability of HUVECs

**Figure 2:**
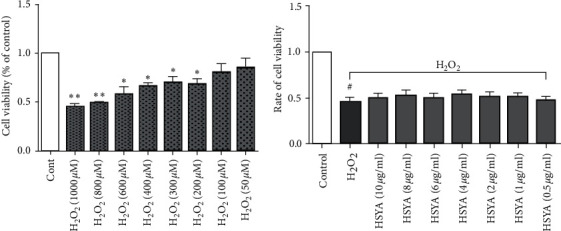
Effects of H_2_O_2_ and HSYA on the viability of HUVECs: (a) HUVECs were treated with different concentrations of H_2_O_2_ (50–1000 *μ*M) for 2 h, and cell viability (OD490) was assessed with an MTT assay; (b) HUVECs were treated with different concentrations of HSYA (0.5–10 *μ*g/ml) for 24 h, and cell viability (OD490) was assessed with an MTT assay. *∗p* < 0.05 and *∗∗p* < 0.01 versus control group; ^*#*^*p* < 0.05 versus H_2_O_2_ group.

MTT assay was used to measure the possible effects on cell viability of H_2_O_2_ or HSYA on HUVECs. As presented in Figure 2(a), after 2 h of induction treatment with different concentrations of H_2_O_2_, the results showed that when the H_2_O_2_ concentration was greater than 200 *μ*M, the cell survival rate was significantly lower than the normal group (*p* < 0.05). Therefore, in combination with the reference results, we chose 200 *μ*M as the H_2_O_2_ model induction concentration.

The data showed that the cell viability after treatment with HSYA at a concentration of 0.5–10 *μ*g/ml was improved to varying degrees compared with the H_2_O_2_ model group (Figure 2(b)). Therefore, in all subsequent experiments, the two most significant concentrations of 8 *μ*g/ml and 4 *μ*g/ml were used.

### 3.2. Effects of HSYA on Cell Proliferation Rate

**Figure 3:**
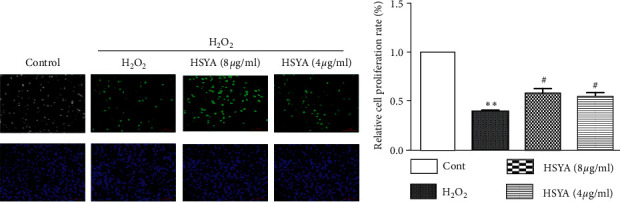
Effects of HSYA on H_2_O_2_-induced HUVEC cell proliferation rate changes: (a) the rate of cell proliferation in HUVECs was detected using an EdU cell proliferation assay; (b) HSYA reverses H_2_O_2_-induced HUVECs cell proliferation rate. The experiment was repeated at least three times. Data were shown as the mean ± SEM of three experiments. *∗∗p* < 0.01 versus control group; ^*#*^*p* < 0.05 versus H_2_O_2_ group.

As presented in Figure 3(a), we used Azide 488 fluorescently labeled probes and Hoechst 33342 to stain proliferating cells and all cell nuclei and observed under a fluorescent microscope. The proliferating cells labeled with Alexa Fluor 488 showed bright green fluorescence, while Hoechst 33342 nuclear-stained cells showed blue fluorescence. The EdU staining results showed that the ratio of Azide 488 labeled cells to Hoechst-labeled cells in the H_2_O_2_ group was significantly reduced, and the cell proliferation rate was significantly reduced (*p* < 0.05). In the HSYA treatment groups, the ratio of Azide 488 labeled cells was significantly higher than that of the H_2_O_2_ group, and the cell proliferation rate was significantly increased (*p* < 0.05).

### 3.3. Effects of HSYA on GSH/GSSG Ratio in Cells

**Figure 4:**
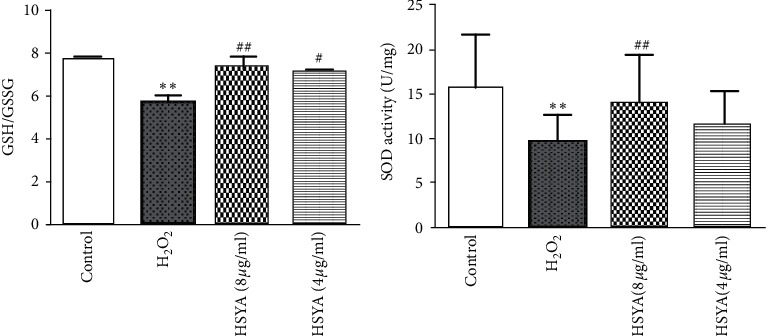
Effect of HSYA on GSH/GSSG ratio and SOD content in cells: (a) the ratio of GSH/GSSG in HUVECs was detected using a GSH and GSSG assay kit; (b) the SOD activity in HUVECs was detected using a SOD assay kit with WST-8. The experiment was repeated at least three times. Data were shown as the mean ± SEM of three experiments. *∗∗p* < 0.01 versus control group; ^*#*^*p* < 0.05 and ^*##*^*p* < 0.01 versus H_2_O_2_ group.

GSH/GSSG ratio is a very useful indicator for measuring the redox status of cells. The occurrence of intracellular oxidative stress will consume a large amount of reduced glutathione, which directly affects the antioxidant level of cells. When the cell is in a peroxidation state, it may cause the reduction of intracellular reduced glutathione to oxidized glutathione; that is, the GSH/GSSG ratio decreases [13].  Figure 4(a) shows that, compared with the control group, the GSH/GSSG ratio of the H_2_O_2_ group was significantly reduced (*p* < 0.01), and the GSH/GSSG ratio increased after HSYA treatments.

### 3.4. Effects of HSYA on SOD Content in Cells

SOD is an important antioxidant enzyme in the body and plays an important role in the body's oxidation and antioxidant balance. We tested the total SOD activity to reflect the redox state of oxidative stress levels in the cells. As presented in Figure 4(b), the results showed that, compared with the control group, the SOD activity of the H_2_O_2_ group was significantly reduced (*P* < 0.01), and the SOD activity was increased after HSYA treatments.

### 3.5. Effect of HSYA on Intracellular ROS Levels

**Figure 5:**
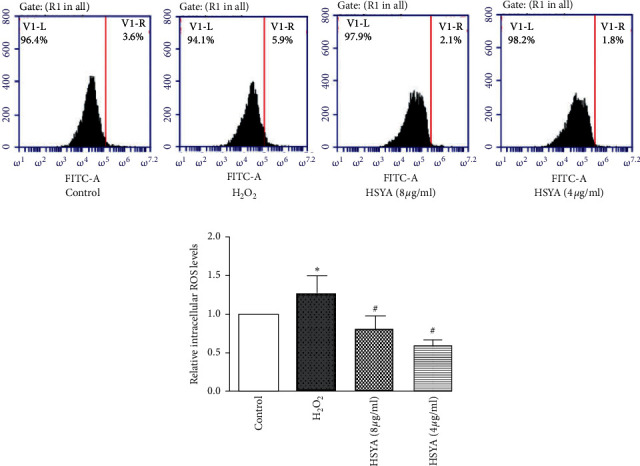
The effect of HSYA on intracellular ROS levels: (a) flow cytometry analysis of intracellular ROS levels in each group; (b) statistical graph of intracellular ROS levels. The experiment was repeated at least three times. Data were shown as the mean ± SEM of three experiments. *∗p* < 0.05 versus control group; ^*#*^*p* < 0.05 versus H_2_O_2_ group.

The unbalanced generation and removal mechanism of reactive oxygen species can easily lead to the accumulation of intracellular ROS, which can induce endothelial cell damage. We tested the levels of ROS in each group of cells. As presented in Figure 5, the results showed that, compared with the control group, the ROS level of the H_2_O_2_ group was significantly increased (*p* < 0.01), and the ROS levels were reduced after HSYA treatments.

### 3.6. Effects of HSYA on Apoptosis-Related Factors BAX and Bcl-2

**Figure 6:**
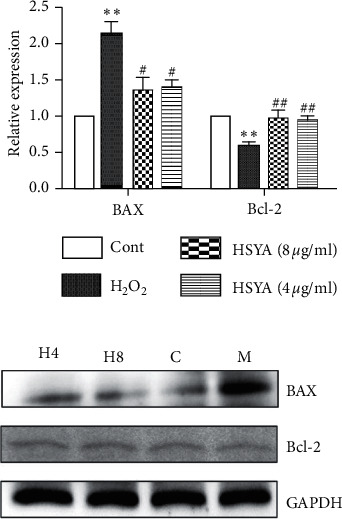
Effects of HSYA on apoptosis-related factors BAX and Bcl-2. HSYA decreased H_2_O_2_-induced BAX expression but increased H_2_O_2_-induced Bcl-2 expression. Data were shown as the mean ± SEM of three experiments. *∗∗p* < 0.01 versus control group; ^*#*^*p* < 0.05 and ^*##*^*p* < 0.01 versus H_2_O_2_ group. C: control; M: H_2_O_2_ group; H8: H_2_O_2_ + HSYA (8 *μ*g/ml) group; H4: H_2_O_2_ + HSYA (4 *μ*g/ml) group.

Bcl-2 and BAX are the major regulators of apoptosis. Bcl-2 is an antiapoptotic gene, and BAX is a proapoptotic gene. The balance of them affects cell apoptosis [14]. As presented in Figure 6, compared with the control group, the expression of BAX protein in the H_2_O_2_ group was significantly increased (*p* < 0.05), and the expression of Bcl-2 protein was significantly decreased (*p* < 0.05), indicating that the H_2_O_2_ group promoted the occurrence of cell apoptosis by upregulating the expression of BAX and inhibiting the expression of Bcl-2. Compared with the H_2_O_2_ group, the expression of BAX protein in the HSYA-treated group was significantly decreased (*p* < 0.05), and Bcl-2 protein expression was significantly increased (*p* < 0.05), indicating that HSYA can inhibit the expression of apoptotic factors in a dose-dependent manner.

### 3.7. Effects of HSYA on AKT and PTEN Protein Expression

**Figure 7:**
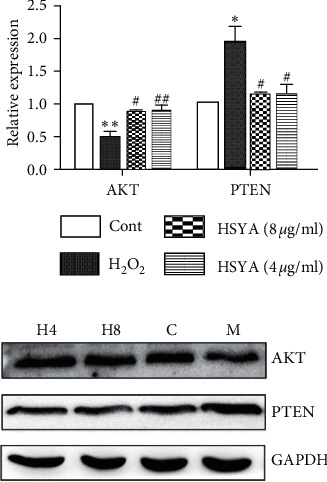
Effects of HSYA on AKT and PTEN protein expression. HSYA decreased H_2_O_2_-induced PTEN expression but increased H_2_O_2_-induced AKT expression. Data were shown as the mean ± SEM of three experiments. *∗p* < 0.05 and *∗∗p* < 0.01 versus control group; ^*#*^*p* < 0.05 and ^*##*^*p* < 0.01 versus H_2_O_2_ group. C: control; M: H_2_O_2_ group; H8: H_2_O_2_ + HSYA (8 *μ*g/ml) group; H4: H_2_O_2_ + HSYA (4 *μ*g/ml) group.

AKT is a key factor that inhibits apoptosis, and PTEN causes apoptosis by inhibiting the activation of AKT. As presented in Figure 7, compared with the control group, the expression of AKT protein in the H_2_O_2_ group was significantly reduced (*p* < 0.05), and the expression of PTEN protein was significantly increased (*p* < 0.05). After HSYA treatments, the expression of AKT protein was significantly increased (*p* < 0.05), and the expression of PTEN protein was significantly reduced (*p* < 0.05) and consistent with previous experimental results.

## 4. Discussion

Endothelial cells play an important role in human health, and the cellular structure and dysfunction caused by oxidative damage were the inducements for many cardiovascular and cerebrovascular diseases [1]. Studies have shown that oxidative damage to endothelial cells was the common pathological basis of various cardiovascular and cerebrovascular diseases such as atherosclerosis, hypertension, and thrombosis [15, 16]. Therefore, it was of great significance to treat cardiovascular and cerebrovascular diseases by inhibiting the occurrence of cellular oxidative damage. Hydroxysafflor yellow A (HSYA) is the main active ingredient of the Chinese medicine safflower plant as a clinical treatment for cardiovascular and cerebrovascular diseases, and our results showed that HSYA can protect endothelial cells by inhibiting the occurrence of oxidative damage.

In the present study, we explored the role of HSYA on H_2_O_2_-induced oxidative damage in HUVECs, and the results showed that treatment with HSYA significantly improved the cell survival and proliferation rate of H_2_O_2_-induced HUVECs oxidative damage, increased the content of reduced glutathione (GSH) in cells, reduced the intracellular ROS level, upregulated the expression of PTEN and BAX, downregulated the expression of AKT and Bcl-2, and inhibited the oxidative damage and even apoptosis of H_2_O_2_-induced HUVECs.

GSH is the most abundant intracellular antioxidant thiol. It is involved in the redox balance of cells and is the key to oxidant-mediated injury defense mechanisms against toxic agents and oxidant-mediated injury [17, 18]. GSH metabolism is tightly regulated in the cell and has also been implicated in redox signaling [19, 20]. The ratio of GSH/oxidized GSH (GSSG) is a very useful indicator to measure the cell's redox status, and the change in the ratio can also directly affect the related signal pathways [21, 22]. Our studies have found that the effect of HSYA at a suitable concentration may increase the intracellular GSH content and significantly increase the GSH/GSSG ratio and mitigation of oxidative stress in HUVECs induced by H_2_O_2_. Studies have shown that an imbalance in GSH was observed in a wide range of pathologies, including cancer, neurodegenerative diseases, and aging [23]. However, whether HSYA can affect the related pathological phenomena by stabilizing the intracellular GSH content or changing the GSH/GSSG ratio is worth further research.

Superoxide dismutase (SOD) is an important metalloenzyme widely existing in various organisms, which can specifically remove superoxide anions and protect the body from oxidative damage. Therefore, it is inseparable from the occurrence and development of many diseases [24]. SOD is frequently used to monitor the treatment of secondary peroxidative injury and the effects of free radical scavenging drugs caused by important organ injuries or surgical treatment, which has an important reference value for tracking the efficacy of free radical scavenging treatment. In this study, we measured the total SOD activity in cells, and it was found that HSYA can relieve the oxidative damage of H_2_O_2_-induced HUVECs by increasing the unit value of SOD enzyme activity in cells.

Besides, reactive oxygen species (ROS) is a free radical in all vascular cells, and they play a key role in regulating various cell functions and biological processes [25]. Although essential for vascular homeostasis, uncontrolled production of ROS has been shown an important effect on vascular endothelial damage [8]. In the study, we carried out flow cytometry of intracellular ROS levels, which confirmed that HSYA reduced the oxidative damage of endothelial cells by regulating the intracellular ROS homeostasis environment, although the data did not show better significant differences.

Oxidative stress is the main factor of oxidative damage to endothelial cells, and oxidative damage to endothelial cells will further induce the occurrence of apoptosis. Intracellular apoptosis-related proteins Bcl-2 and BAX are the two main members of the Bcl-2 protein family, which is essential during the process of apoptosis [26]. In particular, Bcl-2 is the major antiapoptotic protein, which binds to the proapoptotic protein BAX and forms a heterodimer on the outer membrane of mitochondria. This reduces the release of caspase from the mitochondria, which can inhibit cell apoptosis [27]. PTEN was the first tumor suppressor gene with dual-phosphatase activity, which was involved in numerous physiological and pathological processes of the body [28] and was extensively distributed in normal tissue [29]. AKT is a Ser/Thr kinase that regulates cell survival, proliferation, and angiogenesis via phosphorylation of diverse downstream proteins [30, 31]. The literature indicated that activated AKT serves as a transcription factor that regulates downstream substrates and facilitates cellular proliferation [32]. In addition, PTEN prevented the activation of AKT by dephosphorylation, aggravating the apoptosis of cells [33]. Our study indicated that HSYA can effectively inhibit the expression of PTEN protein, increase the expression of AKT protein, and slow the apoptosis of endothelial cells. The mechanism may be related to AKT/PTEN-related signaling pathways.

## 5. Conclusions

Our research showed that HSYA inhibited the oxidative stress of HUVECs induced by H_2_O_2_ and significantly improved cell apoptosis. As an effective monomer component of Chinese herbal medicine, HSYA has provided a certain experimental and theoretical basis for Chinese medicine-assisted targeted therapy of cardiovascular diseases by studying its antioxidant effect.

## Data Availability

The data used to support the findings of this study are available from the corresponding author upon request.
